# Trends in clinical encounters and management for infertility among women attending Australian general practice: a national longitudinal study using MedicineInsight, 2011 to 2021

**DOI:** 10.1136/bmjopen-2024-085149

**Published:** 2025-01-30

**Authors:** Renae C Fernandez, Vivienne Moore, Jacqueline Boyle, Alice R Rumbold, Michael Davies, Danielle Mazza, Luke E Grzeskowiak

**Affiliations:** 1 Robinson Research Institute, The University of Adelaide, Adelaide, South Australia, Australia; 2 Discipline of Obstetrics and Gynaecology, Adelaide Medical School, The University of Adelaide, Adelaide, South Australia, Australia; 3 School of Public Health, The University of Adelaide, Adelaide, South Australia, Australia; 4 Eastern Health Clinical School, Monash University, Box Hill, Victoria, Australia; 5 Department of General Practice, Monash University, Clayton, Victoria, Australia; 6 South Australian Health and Medical Research Institute, Adelaide, South Australia, Australia; 7 SPHERE NHMRC Centre of Research Excellence, Department of General Practice, Monash University, Clayton, Victoria, Australia; 8 College of Medicine and Public Health, Flinders University, Adelaide, South Australia, Australia

**Keywords:** Primary Health Care, Subfertility, Clinical Decision-Making, EPIDEMIOLOGIC STUDIES

## Abstract

**Objective:**

To examine longitudinal trends in infertility management in women attending general practice.

**Design:**

Cohort study using the national general practice dataset, MedicineInsight.

**Setting:**

Australian general practice.

**Intervention(s):**

Not applicable.

**Participants:**

The cohort included 2 552 339 women aged 18–49 years with one or more general practice clinical encounters between January 2011 and December 2021.

**Primary and secondary outcome measures(s):**

The primary outcome assessed was the proportion of women who had a clinical encounter related to infertility, stratified by year and age group. Second, the proportions of women receiving relevant clinical management actions, including selected pathology tests, imaging ordered and selected medications, were calculated. Univariable logistic regression analyses compared the likelihood of women having a documented clinical encounter related to infertility and receiving selected management actions based on individual characteristics. We also examined practice-level variation in the proportion receiving selected management for infertility by stratifying proportions based on practice site.

**Results:**

A total of 2 552 339 women had one or more clinical encounters with their general practitioner (GP) between January 2011 and December 2021, of which 27 671 (1.1%) had a clinical encounter related to infertility management. The rate of infertility encounters increased from 3.4 per 1000 in 2011 to 5.7 per 1000 in 2021. Over episodes of care, half (50.9%) of women presenting for an infertility encounter had at least one specified pathology test, and almost a quarter (23.1%) had a specified imaging test. A relatively small proportion of infertility encounters (5.4%) resulted in prescribing of a selected infertility medication by the GP.

Large variation in clinical management (pathology, imaging and medication prescribing) was evident according to both individual characteristics and also at the clinical-practice level. Factors associated with increased likelihood of being provided infertility medications included younger age, holding a Commonwealth concession card (indicating low income), lower socioeconomic status and living outside a major city.

**Conclusions:**

Clinical encounters related to infertility are increasing in primary care, with large variation evident in corresponding clinical management. These findings support the development of clinical practice guidelines to enhance standardised and equitable approaches towards the management of infertility in primary care.

**STRENGTHS AND LIMITATIONS OF THIS STUDY**

- Use of a large, high-quality general practice dataset containing longitudinal individual-level data allows examination of the entire episode of care by capturing all encounters relating to the same individual over a period of time at the same general practice.
- Referrals made to fertility clinics or specialists could not be examined and it is assumed that the selected pathology tests and imaging ordered were related to the infertility encounter occurring on the same date.
- Patient data are not linked across different general practices, therefore double counting of individuals who present at more than one general practice is possible.
- This study was conducted within the context of the Australian health care system; thus the findings may not be generalisable outside this context.

## Introduction

 Infertility represents a significant health problem for women and men worldwide. Over their lifetime, one in six couples will experience infertility,^1^ defined as the inability to conceive a pregnancy after 12 months of regular unprotected sexual intercourse.^2^ Infertility may be related to either male or female factors in isolation or in combination, such as low semen count, poor sperm motility, tubal factors or anovulatory infertility where polycystic ovary syndrome (PCOS) is often implicated. Approximately 15% of cases remain of unknown aetiology.^3^ The age-related decline in fertility that occurs in women from about 35 years is another major contributor.^4^ The incidence of infertility has increased over time due to delayed family formation and increasing maternal age at first birth (current average of 30 years in Australia) and the trend for increasing body mass in the population which are linked to anovulation, miscarriage and adverse pregnancy outcomes for mother and child.^5 6^ Parenthood is a life-defining experience and hence infertility is often accompanied by stress and impaired physical, emotional and psychological well-being.^7^

It is estimated that 50% of women who experience difficulty conceiving will seek medical assistance.^8 9^ Similar to many countries internationally, general practitioners (GPs) in Australia are the major providers of primary healthcare services and are the first point of contact for infertility care. Patient costs to access Australian GP services are completely or partially covered by the universal healthcare system, Medicare. GPs may make initial investigations of fertility concerns including ordering pathology tests and imaging, manage reproductive symptoms, and are responsible for determining need for referral to specialist healthcare services, such as gynaecologists and fertility clinics providing assisted reproductive technology (ART) treatment. Some GPs with a special interest in women’s health may also complete additional relevant training to enable them to manage infertility with non-invasive treatments. Although there are no Australian general practice guidelines specifically for the management of infertility, accessible guidelines do exist in other countries.^10^ Further, the development of the international evidence-based guideline for the assessment and management of PCOS has broadened the scope of practice for GPs in the management of female infertility.^11^

Despite being the first point of contact for women who are experiencing infertility, there is little longitudinal data on the characteristics of those who seek care or the infertility management activities of GPs. Existing evidence comes from the Bettering the Evaluation and Care of Health (BEACH) study, which contains data from 100 consultations recorded by 1000 GPs who are randomly selected yearly.^12^ Analyses of these data indicated that over the period 2000–2016, 5.8 per 1000 encounters for women aged 18–49 years were for infertility care, an increase from 1.3 per 1000 encounters in 1998–2004.^13 14^ Attendance at a general practice for infertility care was more common among women with higher socioeconomic status, living in major cities, not receiving social welfare assistance, and being from a non-English speaking background.^13^ Among 2118 women presenting for infertility management, 42.9% of encounters involved advice/education/counselling, 42.1% referral to a fertility clinic or specialist, 25.7% pathology testing, 9.3% imaging and 0.8% prescription of infertility medication.^13^ However, a key limitation of the BEACH study is that it was cross-sectional, making it difficult to distinguish between the first, second, third, etc encounters for the same problem. Thus, analysis of infertility management over an entire course of care at an individual level was not possible, limiting the assessment of variability in treatment planning, and therefore, future guideline development. Further, examination of practice-level variation has not been performed in previous studies.

The current study aims to identify and examine longitudinal trends in infertility management among women attending general practice and to describe practice-level variation in the proportion receiving selected management for infertility.

## Methods

### Study design, setting and data source

This was a cohort study assembled using data from the National Prescribing Scheme (NPS) MedicineWise MedicineInsight dataset. The study period spanned 1 January 2011 to 31 December 2021. MedicineInsight is a large-scale, national general practice dataset established by NPS MedicineWise with core funding from the Australian Government Department of Health. The MedicineInsight dataset has been described in detail elsewhere.^15^ In summary, MedicineInsight uses third-party extraction tools (GRHANITE and Precedence Health Care’s cdmNet) which extract, de-identify and securely transmit patient data from participating practices’ clinical information systems, such as Best Practice and Medical Director, to a secure data repository. The extraction tool collects incremental data regularly, allowing the development of a longitudinal dataset in which individuals within each practice can be tracked over time. The MedicineInsight dataset collects data on individual demographics, practice encounters (not including progress notes), diagnoses, prescribed medication and pathology tests, and selected free text data. MedicineInsight contains electronic health records from approximately 2700 GPs and 662 general practices across Australia (8.2% of all Australian practices).^4^ The characteristics of MedicineInsight patients have been previously demonstrated to be nationally representative of the Australian population.^16^

In Australia, patient costs for many healthcare services are completely or partially covered by the universal healthcare system, Medicare. This system provides a universal subsidy for consulting GPs, for blood tests and imaging and specialists, but there is often an additional out-of-pocket cost (copayment) that is determined by the individual service provider and this is typically larger for services other than those of GPs.

### Study population

For this study, we restricted our analysis to women of reproductive age (18–49 years inclusive). The MedicineInsight programme uses the terms sex and gender interchangeably and presents sex/gender information as a single variable. In line with the language used by the MedicineInsight programme, this paper has used the term ‘women’ to describe the cohort, but it is acknowledged that sex and gender are distinct concepts, and that sex and gender do not automatically align.

### Patient and public involvement

No patients or members of the public were involved in the development of the research question or the outcome measures, or in the design and conduct of the study.

### Outcome

The primary outcome assessed was the proportion of women who had a clinical encounter related to infertility management. Infertility encounters were identified by searching the ‘encounter reason’ field, with the list of selected infertility problems terms provided in online supplemental table S1. Related outcomes included the relevant management actions provided to women during infertility encounters, including selected pathology tests and imaging ordered, and selected medications (ie, metformin, clomifene and letrozole) prescribed to individuals. The selected management actions are defined in online supplemental table S1. These were specifically chosen to compare with previous research undertaken in Australian general practice.^13^

### Covariates

Patient characteristics included age (based on year of birth), remoteness, area-level socioeconomic status (using the socioeconomic indexes for areas, SEIFA), state/territory, Indigenous status, Commonwealth concession card status and smoking status. Remoteness, SEIFA and state/territory were based on patients’ residential postcodes. Remoteness was determined in accordance with the Australian Bureau of Statistics (ABS) Australian Statistical Geography Standard remoteness areas, with 1 being a major city and 5 being a very remote area. Due to small population sizes, data for ‘remote’ and ‘very remote’ were combined. SEIFA was determined according to the ABS Index of Relative Socio-Economic Advantage and Disadvantage codes.^17^ Additional characteristics of the cohort included the presence of relevant comorbidities comprising PCOS, diabetes, asthma, anxiety and history of depression. Individuals were defined as having any of these clinical conditions based on ‘condition flags’ included in the MedicineInsight dataset, with the date of recording preceding the date of their first infertility encounter.^18^

### Statistical analysis

Descriptive statistics (counts and percentages) were used to describe the study population. Annual proportion was calculated based on those who had at least one infertility encounter in a given calendar year divided by the total number who had any clinical encounter in the same year and expressed per 1000 women. The proportion of infertility encounters was further stratified based on age group. The proportions of women for whom selected pathology testing or imaging was ordered, or a selected medication was prescribed, were calculated based on the year of first clinical encounter for infertility and expressed as a percentage. Proportions were calculated separately based on management occurring on the same day as the first documented clinical encounter for infertility or on the same day as any clinical encounter for infertility. We examined practice-level variation in the proportion receiving selected management for infertility by stratifying proportions based on practice site.

We used univariable logistic regression analyses to compare the likelihood of women having a documented clinical encounter related to infertility based on individual characteristics. We also used univariable logistic regression analyses to compare the likelihood of women receiving selected clinical management for infertility according to individual characteristics, reported separately according to the time of the first clinical encounter, or any clinical encounter. Longitudinal trends in the proportion of women receiving selected clinical management for infertility were analysed by including year as a continuous variable in the logistic regression model.

All analyses were based on two-sided p values, with statistical significance defined by p<0.05. The statistical analysis was performed using STATA MP V.17 (Stata), with graphs prepared using R, V.4.3.0 (R Core Team).

## Results

A total of 2 552 339 women aged 18–49 years had one or more clinical encounters with their GP between January 2011 and December 2021, with 27 671 (1.1%) women having one or more clinical encounters related to infertility.

**Figure 1:**
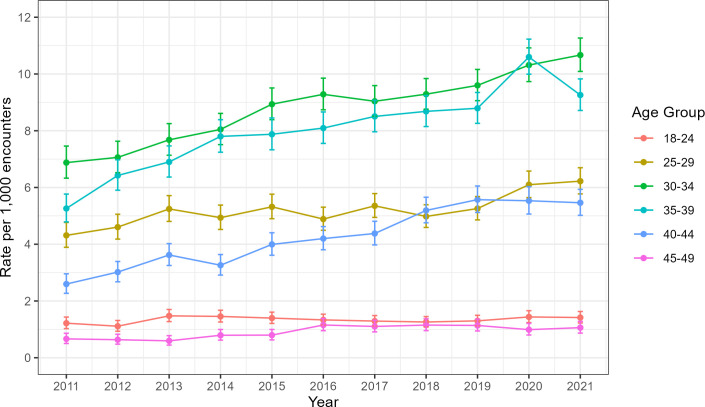
Annual prevalence rate of infertility encounters in general practice among women aged 18–49 according to age group, Australia 2011–2021.

The annual prevalence of clinical encounters related to infertility increased from 3.4 per 1000 women in 2011 to 5.7 per 1000 in 2021. The highest rates were evident among women aged 30–34, with marked increases over time observed among those aged between 30 and 34 (P trend <0.001), 35–39 (P trend <0.001), and 40–44 (P trend <0.001) (figure 1). Over the 10-year period, clinical encounters related to infertility increased by 55%, 75% and 111%, respectively, in these age groups.

Lower rates of infertility encounters were evident among those who had a Commonwealth concession card (indicating low income), were current smokers, lived in regional or remote areas or were Aboriginal and/or Torres Strait Islander (table 1). Recorded comorbidities of asthma, anxiety, depression, and PCOS were each associated with a higher rate of infertility encounters (table 1).

**Table 1:** Characteristics of women presenting for infertility encounters to general practice, Australia 2011–2021

Category	Cases/women	Rate per 1000 (95% CI)	OR (95% CI)
Concession status			
No concession	20 447/1 719 101	11.9 (11.7, 12.1)	Reference
Concession holder	3939/453 092	8.7 (8.4, 9.0)	0.77 (0.74, 0.79)
Not recorded	3285/380 146	8.6 (8.3, 8.9)	0.72 (0.70, 0.75)
Smoking status			
Never smoker	14 631/919 302	15.9 (15.7, 16.2)	Reference
Ex-smoker	5429/254 592	21.3 (20.8, 21.9)	1.35 (1.31, 1.39)
Current smoker	5008/735 205	6.8 (6.6, 7.0)	0.42 (0.41, 0.44)
Not recorded	2603/643 240	4.0 (3.9, 4.2)	0.25 (0.24, 0.26)
Remoteness			
Major city	19268/1 721 793	11.2 (11.0, 11.6)	Reference
Inner/outer regional	7975/765 336	10.4 (10.2, 10.7)	0.93 (0.91, 0.96)
Remote or very remote	305/38 651	7.9 (7.0, 8.8)	0.70 (0.63, 0.79)
Not recorded	123/26 559	4.6 (3.9 to 5.5)	0.41 (0.34 to 0.49)
Socioeconomic status			
Very low	3686/357 529	10.3 (10.0, 10.7)	1.02 (0.98, 1.06)
Low	4778/427 638	11.2 (10.9, 11.5)	1.11 (1.07, 1.15)
Middle	5780/492 232	11.7 (11.4, 12.1)	1.17 (1.13, 1.21)
High	6269/551 643	11.4 (11.1, 11.7)	1.13 (1.09, 1.17)
Very high	7035/696 993	10.1 (9.9, 10.3)	Reference
Indigenous status			
Aboriginal and/or TSI	600/61 680	9.7 (9.0, 10.5)	0.75 (0.69, 0.82)
Non-Indigenous	21 717/1 686 254	12.9 (12.7, 13.1)	Reference
Not recorded	5354/804 405	6.7 (6.5, 6.8)	0.51 (0.50, 0.53)
Asthma			
No	25 958/2 426 100	10.7 (10.6, 10.8)	Reference
Yes	1713/126 239	13.6 (12.9, 14.2)	1.27 (1.21, 1.34)
Anxiety			
No	26 004/2 421 433	10.7 (10.6, 10.9)	Reference
Yes	1667/130 906	12.7 (12.1, 13.4)	1.19 (1.13, 1.25)
Depression			
No	25 366/2 367 985	10.7 (10.6, 10.8)	Reference
Yes	2305/184 354	12.5 (12.0, 13.0)	1.17 (1.12, 1.22)
PCOS			
No	26 504/2 526 708	10.5 (10.4, 10.6)	Reference
Yes	1167/25 631	45.5 (43.0, 48.2)	4.50 (4.24, 4.78)
Diabetes			
No	27 494/2 533 784	10.9 (10.7, 11.0)	Reference
Type1 diabetes mellitus	54/5448	9.9 (7.5, 12.9)	0.91 (0.70, 1.19)
Typte 2 diabetes mellitus	123/13 107	9.4 (7.8, 11.2)	0.86 (0.72, 1.03)

PCOSpolycystic ovary syndromeTSITorres Strait Islander

**Figure 2:**
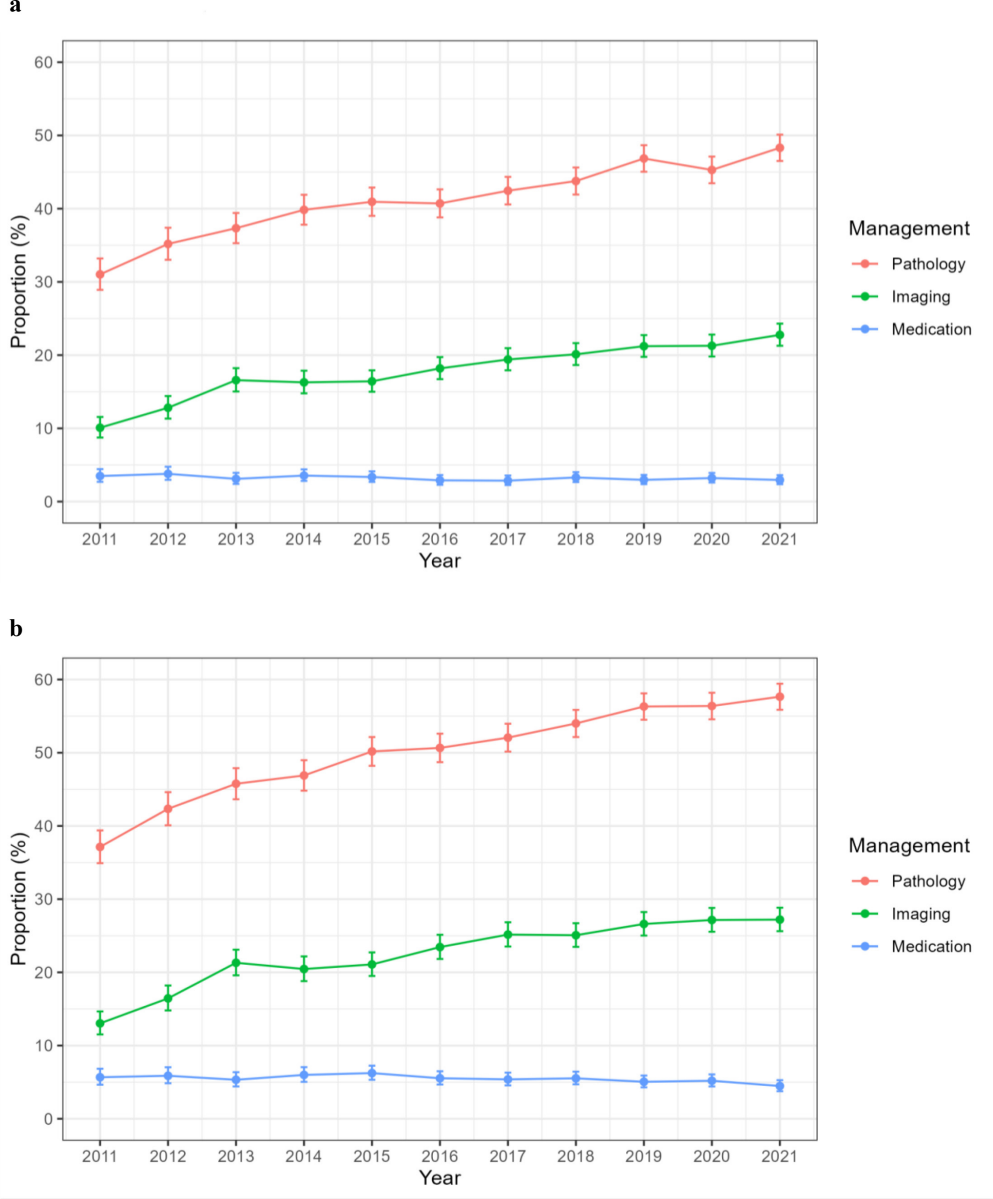
Proportion of individuals presenting for infertility clinical encounters where the general practitioner provided specific management, Australia 2011–2021. (a) First clinical encounter, (b) any clinical encounter.

At the time of the first encounter, the proportion of women who received at least one specified pathology test increased from 31.0% in 2011 to 48.3% in 2021 (P trend <0.001), and the proportion who received a specified imaging test increased from 10.1% in 2011 to 22.8% in 2021 (P trend <0.001) (figure 2a). In contrast, the proportion of women attending encounters resulting in the provision of a selected medication declined slightly from 2011 (3.5%) to 2021 (3.0%) (P trend=0.011). Similar patterns emerged when considering clinical management occurring during any infertility encounter (figure 2b).

Overall, half (50.9%) of women presenting for an infertility encounter had at least one specified pathology test, and almost a quarter (23.1%) had a specified imaging test. A relatively small proportion of infertility encounters (5.4%) resulted in a selected medication being prescribed by the GP. GP practices differed in the frequency with which patients were referred for imaging or pathology tests, and were prescribed medications. The extent of variation was large, ranging from 0% to 81.8% for pathology, 0% to 57.8% for imaging, and 0% to 52.2% for medications (online supplemental figure 1).

While a relatively small proportion of women were prescribed medication during their course of care with the GP, younger age, having a Commonwealth concession card, living in a regional or remote area, lower socioeconomic status, being Aboriginal and/or Torres Strait Islander, and having PCOS or diabetes were all associated with increased likelihood of being prescribed specified infertility medications (table 2). Between 2011 and 2021, the proportion of individuals prescribed clomifene declined from 3.4% to 0.7% (P trend <0.001), whereas an increase was observed for letrozole (0%–0.8%, P trend <0.001) (online supplemental table S2). Prevalence of metformin remained stable at 3.1% (P trend=0.162).

**Table 2:** Provision of infertility medications to women attending infertility-related encounter to Australian general practice between January 2011 and December 2021

	First clinical encounter		Any clinical encounter	
**Category**	**Rate per 100**(**95% CI**)	**OR**(**95% CI**)	**Rate per 100**(**95% CI**)	**OR**(**95% CI**)
Age group				
18–24	4.5 (3.7, 5.6)	1.43 (1.12, 1.82)	7.9 (6.7, 9.2)	1.50 (1.24, 1.82)
25–29	4.6 (4.1, 5.2)	1.44 (1.21, 1.71)	7.8 (7.1, 8.5)	1.48 (1.30, 1.69)
30–34	3.2 (2.9, 3.6)	Reference	5.4 (4.9, 5.9)	Reference
35–39	2.4 (2.1, 2.8)	0.74 (0.61, 0.89)	4.1 (3.6, 4.6)	0.75 (0.64, 0.87)
40–44	2.0 (1.6, 2.6)	0.62 (0.47, 0.81)	3.7 (3.1, 4.4)	0.68 (0.55, 0.83)
45–49	1.5 (0.8, 2.7)	0.47 (0.26, 0.86)	2.7 (1.6, 4.1)	0.48 (0.30, 0.76)
Concession status				
No concession	2.8 (2.6, 3.0)	Reference	4.8 (4.5, 5.1)	Reference
Concession holder	4.6 (4.0, 5.3)	1.59 (1.35, 1.88)	8.3 (7.4, 9.2)	1.72 (1.52, 1.96)
Not recorded	4.1 (3.4, 4.8)	1.49 (1.23, 1.81)	5.8 (5.1, 6.7)	1.23 (1.04, 1.44)
Smoking status				
Never smoker	3.0 (2.7, 3.2)	Reference	5.2 (4.9, 5.6)	Reference
Ex-smoker	3.7 (3.2, 4.3)	1.27 (1.07, 1.51)	5.6 (5.0, 6.2)	1.08 (0.94, 1.24)
Current smoker	3.4 (2.9, 3.9)	1.16 (0.97, 1.39)	5.7 (5.1, 6.4)	1.10 (0.96, 1.27)
Not recorded	3.1 (2.4, 3.8)	1.04 (0.82, 1.33)	5.8 (4.9, 6.8)	1.12 (0.94, 1.34)
Remoteness				
Major city	2.9 (2.7, 3.2)	Reference	5.1 (4.8, 5.4)	Reference
Inner/outer regional	3.8 (3.4, 4.2)	1.31 (1.14, 1.51)	6.2 (5.7, 6.8)	1.24 (1.11, 1.39)
Remote or very remote	5.3 (3.0, 8.4)	1.84 (1.11, 3.07)	7.9 (5.1, 11.5)	1.60 (1.05, 2.44)
Not recorded	2.4 (0.5, 7.0)	0.83 (0.26, 2.62)	3.3 (0.9, 8.1)	0.63 (0.23, 1.71)
Socioeconomic status				
Very low	4.0 (3.4, 4.7)	2.33 (1.83, 2.96)	6.8 (6.0, 7.7)	2.31 (1.91, 2.78)
Low	3.6 (3.1, 4.2)	2.08 (1.64, 2.62)	5.7 (5.1, 6.4)	1.90 (1.58, 2.28)
Middle	4.1 (3.6, 4.7)	2.38 (1.92, 2.97)	7.6 (6.9, 8.3)	2.57 (2.18, 3.04)
High	3.1 (2.7, 3.6)	1.77 (1.41, 2.22)	5.1 (4.6, 5.7)	1.70 (1.42, 2.02)
Very high	1.8 (1.5, 2.1)	Reference	3.1 (2.7, 3.5)	Reference
Indigenous status				
Aboriginal and/or TSI	6.8 (5.0, 9.2)	2.20 (1.58, 3.04)	9.7 (7.4, 12.3)	1.79 (1.36, 2.37)
Non-Indigenous	3.2 (3.0, 3.5)	Reference	5.6 (5.3, 5.9)	Reference
Not recorded	2.6 (2.2, 6.1)	0.80 (0.67, 0.97)	4.2 (3.6, 4.7)	0.73 (0.63, 0.83)
Asthma				
No	3.2 (3.0, 3.4)	Reference	5.4 (5.1, 5.7)	Reference
Yes	3.6 (2.8, 4.6)	1.15 (0.88, 1.50)	5.8 (4.8, 7.1)	1.09 (0.88, 1.34)
Anxiety				
No	3.2 (3.0, 3.4)	Reference	5.5 (5.2, 5.7)	Reference
Yes	3.2 (2.4, 4.1)	1.00 (0.75, 1.32)	5.2 (4.2, 6.3)	0.94 (0.75, 1.18)
Depression				
No	3.1 (2.9, 3.4)	Reference	5.4 (5.1, 5.7)	Reference
Yes	3.9 (3.2, 4.8)	1.26 (1.01, 1.57)	6.1 (5.2, 7.2)	1.15 (0.96, 1.37)
PCOS				
No	2.8 (2.6, 3.0)	Reference	4.8 (4.6, 5.1)	Reference
Yes	12.8 (10.9, 14.8)	5.14 (4.26, 6.19)	18.1 (15.9, 20.4)	4.31 (3.67, 5.05)
Diabetes				
No	3.1 (2.9, 3.4)	Reference	5.3 (5.1, 5.6)	Reference
Type 1 diabetes mellitus	9.3 (3.1, 20.3)	3.15 (1.25, 7.93)	9.3 (3.1, 20.3)	1.81 (0.72, 4.54)
Type 2 diabetes mellitus	13.0 (7.6, 20.3)	4.62 (2.72, 7.85)	23.6 (16.4, 32.1)	5.47 (3.59, 8.32)

PCOS, polycystic ovary syndrome; TSI, Torres Strait Islander

Older age, previous or current smoking, and the presence of comorbidities of asthma, anxiety, depression or PCOS were all associated with a lower likelihood of having a specified imaging request ordered on the same day as the first infertility encounter (table 3). Similar associations were seen with respect to the likelihood of specified pathology investigations, with the addition that having a Commonwealth concession card was also associated with a lower likelihood of specified pathology investigations (online supplemental table S3). The vast majority (>98%) of specified imaging requests involved pelvic ultrasounds, which increased in prevalence from 12.7% in 2011 to 27% in 2021 (P trend <0.001) (online supplemental table S4). The most common pathology requests involved thyroid function (38.2%) and haemoglobin (36.2%), as well as reproductive hormones (luteinizing hormone, follicle stimulating hormone and progesterone) (27.9%–31%) (online supplemental table S5). Between 2011 and 2021, the largest increases in pathology requests were observed for haemoglobin A1C (14-fold: 0.7%–10.1%, P trend <0.001) and antimullerian hormone (7-fold: 2.2%–15.3%, P trend <0.001).

**Table 3:** Imaging requests among women attending infertility-related encounter to Australian general practice between January 2011 and December 2021

	First clinical encounter		Any clinical encounter	
**Category**	**Rate per 100**(**95% CI**)	**OR**(**95% CI**)	**Rate per 100**(**95% CI**)	**OR**(**95% CI**)
Age group				
18–24	22.9 (21.0, 24.8)	1.23 (1.09, 1.38)	27.7 (25.7, 29.7)	1.17 (1.05, 1.31)
25–29	23.1 (22.0, 24.2)	1.25 (1.15, 1.35)	28.5 (27.3, 29.7)	1.22 (1.13, 1.31)
30–34	19.5 (18.6, 20.3)	Reference	24.7 (23.8, 25.6)	Reference
35–39	15.9 (15.0, 16.8)	0.78 (0.72, 0.85)	20.7 (19.7, 21.6)	0.79 (0.74, 0.86)
40–44	11.5 (10.4, 12.6)	0.54 (0.48, 0.60)	14.8 (13.6, 16.1)	0.53 (0.48, 0.59)
45–49	9.8 (7.7, 12.2)	0.45 (0.35, 0.58)	12.6 (10.2, 15.2)	0.44 (0.35, 0.55)
Concession status				
No concession	19.0 (18.4, 19.5)	Reference	24.0 (23.4, 24.6)	Reference
Concession holder	18.6 (17.2, 19.6)	1.00 (0.92, 1.09)	23.2 (21.9, 24.6)	1.00 (0.93, 1.09)
Not recorded	14.3 (13.1, 15.5)	0.71 (0.64, 0.79)	17.4 (16.2, 18.8)	0.67 (0.61, 1.04)
Smoking status				
Never smoker	19.5 (18.9, 20.1)	Reference	24.9 (24.2, 25.6)	Reference
Ex-smoker	15.3 (14.3, 16.3)	0.74 (0.68, 0.81)	19.3 (18.3, 20.4)	0.72 (0.67, 0.78)
Current smoker	18.1 (17.0, 19.1)	0.91 (0.84, 0.99)	22.4 (21.3, 23.6)	0.87 (0.81, 0.94)
Not recorded	18.5 (17.0, 20.1)	0.94 (0.84, 1.04)	22.3 (20.7, 23.9)	0.86 (0.78, 0.95)
Remoteness				
Major city	17.8 (17.2, 18.3)	Reference	22.7 (22.1, 23.3)	Reference
Inner/outer regional	19.7 (18.8, 20.6)	1.13 (1.06, 1.21)	24.2 (23.3, 25.1)	1.09 (1.02, 1.15)
Remote or very remote	20.3 (16.0, 25.3)	1.18 (0.89, 1.57)	24.9 (20.2, 30.2)	1.13 (0.87, 1.47)
Not recorded	13.0 (7.6, 20.3)	0.69 (0.41, 1.17)	16.3 (10.2, 24.0)	0.66 (0.41, 1.07)
Socioeconomic status				
Very low	18.2 (17.0, 19.5)	1.17 (1.06, 1.30)	22.5 (21.2, 23.9)	1.14 (1.03, 1.25)
Low	20.5 (19.4, 21.7)	1.36 (1.24, 1.49)	25.5 (24.3, 26.8)	1.34 (1.23, 1.46)
Middle	18.7 (17.7, 19.7)	1.21 (1.10, 1.32)	24.2 (23.1, 25.3)	1.25 (1.15, 1.36)
High	19.0 (18.1, 20.0)	1.24 (1.13, 1.35)	23.9 (22.8, 25.0)	1.23 (1.13, 1.33)
Very high	16.0 (15.1, 16.9)	Reference	20.4 (19.4, 21.3)	Reference
Indigenous status				
Aboriginal and/or TSI	20.7 (17.5, 24.1)	1.15 (0.94, 1.41)	26.2 (22.7, 29.9)	1.16 (0.96, 1.39)
Non-Indigenous	18.4 (17.9, 18.9)	Reference	23.5 (22.9, 24.0)	Reference
Not recorded	17.7 (16.6, 18.7)	0.95 (0.88, 1.41)	21.4 (20.3, 22.5)	0.89 (0.83, 1.39)
Asthma				
No	18.5 (18.0, 19.0)	Reference	23.4 (22.9, 23.9)	Reference
Yes	15.8 (14.1, 17.6)	0.83 (0.72, 0.94)	19.3 (17.4, 21.2)	0.78 (0.69, 0.88)
Anxiety				
No	18.6 (18.1, 19.1)	Reference	23.4 (22.9, 23.9)	Reference
Yes	13.6 (12.0, 15.4)	0.69 (0.60, 0.80)	18.7 (16.8, 20.6)	0.75 (0.66, 0.85)
Depression				
No	18.5 (18.0, 19.0)	Reference	23.4 (22.9, 23.9)	Reference
Yes	15.9 (14.4, 17.4)	0.83 (0.74, 0.93)	20.4 (18.7, 22.1)	0.84 (0.75, 0.93)
PCOS				
No	18.5 (18.1, 19.0)	Reference	23.4 (22.9, 23.9)	Reference
Yes	13.3 (11.4, 15.4)	0.67 (0.57, 0.80)	16.9 (14.8, 19.2)	0.66 (0.57, 0.78)
Diabetes				
No	18.4 (17.9, 18.8)	Reference	23.2 (22.7, 23.7)	Reference
Type 1 diabetes mellitus	9.3 (3.1, 20.3)	0.45 (0.18, 1.14)	11.1 (4.2, 22.6)	0.41 (0.18, 0.97)
Type 2 diabetes mellitus	14.6 (8.9, 22.1)	0.76 (0.46, 1.26)	19.5 (12.9, 27.6)	0.80 (0.51, 1.26)

PCOS, polycystic ovarian syndrome; TSI, Torres Strait Islander

## Discussion

In this cohort study involving MedicineInsight data, annual primary care encounters for infertility increased by 70% over the period of study, 2011–2021. A higher prevalence of infertility encounters was associated with women aged over 30 years and the presence of comorbidities, whereas infertility encounters were less likely among those with lower socioeconomic status or residing in regional or remote locations. Half of women presenting for an infertility encounter had at least one specified pathology test, and almost one quarter had a specified imaging test. A relatively small proportion of infertility encounters (5.4%) involved the provision of a selected infertility medication. Large variability in clinical management was evident according to both individual characteristics and also at the practice level. Provision of infertility medication varied by individual characteristics to a greater degree than other clinical management options (pathology and imaging). Increased likelihood of being prescribed infertility medications included younger age, holding a Commonwealth concession card (indicating low income), lower socioeconomic status and living outside of a major city.

The annual prevalence of clinical encounters related to infertility increased 1.7-fold from 2011 to 2021. As expected, the highest increase was observed among women over 30. This corresponds with trends in increasing age of women at first pregnancy (average of 28.4 years in 2011, average of 29.7 years in 2021) and increasing age-related infertility.^19^ Using BEACH data, Chambers *et al* reported a similar increase in annual prevalence of infertility-related clinical encounters among Australian women, 1.6-fold from 2000 to 2016, with a peak in the 35–39 years age group.^13^ A large UK population-based cohort study of fertility problems in primary care reported the highest occurrence of fertility presentations among the 30–34 years age group.^20^

Lower rates of infertility encounters among those holding a Commonwealth concession card, current smokers and those living in regional and remote areas may reflect lower health literacy, lower rates of access to healthcare, and difficulty navigating services more generally.^21^ The over-riding implication for health services organisation for these patients is that provision of primary healthcare services should meet overall needs, of which fertility treatment is just one component. Earlier family formation in these socioeconomic groups could mean there is less age-related infertility.^22 23^ Lower rates of GP encounters for infertility among those living in regional and remote areas could reflect a lack of access due to lower supply of health services in these areas. These patterns largely reflect those of previous studies.^13 14^

Overall, half of women presenting to their GP for infertility care had a least one pathology test, and almost a quarter had a specified imaging test. Both referral for pathology testing and imaging showed considerable variation across practices, 0%–81.8% and 0%–57.3%, respectively. Two previous studies of BEACH data reported 25.7% and 27.3% pathology testing,^13 14^ whereas a third study reported 88.4% pathology testing.^24^ Imaging requested in previous studies showed less variability, overall lower than in the current study, ranging from about 6% to 10% of encounters.^13 14 24^ Differences are likely to reflect the cross-sectional nature of the BEACH data set and changes across time periods of study.

Around 3% of women were prescribed medication during their first infertility care encounter, increasing to around 5%–6% for any infertility encounter. This was relatively stable over time. The proportion of women prescribed medication was higher than reported in the most recent analysis of BEACH data,^13^ but similar to earlier reports.^14 24^ Over the time period of the study, specific trends indicate movement away from prescribing clomifene citrate in favour of letrozole, possibly because of better live birth rates.^3^ Notably, in many states/territories in Australia, the prescribing of clomifene is currently (or has previously been) restricted to certain specialists (eg, endocrinology, obstetrics/gynaecology), whereas no such restrictions are in place for letrozole. Although a universal healthcare insurance system operates in Australia, the patient out-of-pocket costs associated with attending a specialised clinic are considerably more than those associated with GP appointments. Medical specialists are also concentrated in major cities. Patterns in the prescription of specific medications by GPs may, therefore, suggest that medications are prescribed to women who may have difficulty accessing specialist care, that is those with lower socioeconomic status and those living outside major cities.

Women with PCOS and diabetes were also more likely to be prescribed medication. Ovulatory dysfunction and menstrual irregularity are key diagnostic criteria for PCOS,^11^ therefore it is not unexpected that women with PCOS were prescribed medication to stimulate ovulation. Indeed, the diagnosis of PCOS may follow the presentation of disorders related to infertility. The higher rate of prescriptions to younger women may relate to PCOS status, or an attempt to mitigate fertility problems before more intensive investigation and treatment are attempted.^9^ Further, infertility among younger women may be managed in general practice for longer, whereas women who are aged over 35 years and at risk of age-related infertility may be more likely to be referred to specialist health services sooner and proceed without delay to therapy such as in vitro fertilization (IVF).

There was considerable variation in clinical management of infertility at the practice level. This was most evident in the ordering of imaging and pathology. Some variation at the practice level is not unexpected, and likely reflects differences in patient casemix, GP’s experience, therapeutic approach and specialist knowledge/training and other practice-related factors. However, variations by socioeconomic status and remoteness of the patient indicate potential inequalities in access to infertility care. At present there are no formal clinical guidelines for the management of infertility in the general practice setting in Australia. Implementation of clinical guidelines could improve consistency and equity of care provided to patients and provide a more streamlined process for GPs.^25^ The increasing availability and accessibility of infertility treatments, including ART,^26 27^ and demographic trends to increased age at first pregnancy in developed countries, indicate a mounting need for management guidelines in primary care. In countries such as the Netherlands and the UK, where the cost of infertility treatment is similarly subsidised by government, evidence-based guidelines for the management of infertility in primary care have been developed to promote optimal patient care while controlling unnecessary expenditure.^25 28^

This study has a number of strengths including the use of a large, high-quality general practice dataset containing longitudinal individual-level data from 2011 to 2021. Longitudinal individual-level data allow examination of the entire episode of care by capturing all encounters relating to the same individual over a period of time at the same general practice. This approach represents a major enhancement over previous cross-sectional studies that could only capture a single encounter and not the entire course of care in general practice.

Notable limitations of this analysis include the assumption that the selected pathology/imaging was related to the documented encounter reason. We did not examine pathology and imaging history that may have been undertaken for non-infertility reasons. We also do not have data on referrals made to fertility clinics or specialists. However, in countries with existing clinical guidelines, such as the UK and the Netherlands, the recommendation is for basic infertility investigations to be undertaken by GPs prior to referral to specialist care,^25 29^ providing the rationale for this study to investigate initial management in primary care.

While data are recorded at the individual patient level, patient data are not linked across different general practices. It is therefore possible to double count individuals presenting to different general practices. An Australian survey estimated multiple general practice attendance at 28%. However, 85% of these individuals also reported having a usual GP, and attendance at non-usual GPs may be for acute and less complex health concerns.^30^

A notable proportion of data for the covariates of Commonwealth concession card, smoking and Indigenous status was not recorded. These data are not likely to be missing at random. Data entry often relies on GPs selecting ‘yes’ to indicate the presence of the characteristic, thus it is likely that where nothing was recorded it is suggestive of the individual not having the characteristic of interest. Further, the strongest predictor of missing data in the MedicineInsight dataset is the number of clinical encounters with the general practice site, with greater missing data for those with fewer clinical encounters.^31^ As statistical imputation would not be appropriate in this circumstance, we present regression results for a ‘not recorded’ category. Women for whom data on these covariates were not recorded were less likely to attend for an infertility encounter or receive clinical management for infertility. This is not unexpected, as given the sensitivity and complexity of infertility, women may be less inclined to see a new or temporary GP for this purpose.

MedicineInsight uses a non-random sampling process to recruit the practices, however, the distribution of the sample closely resembles figures from the latest Australian census.^15^ Lastly, it should also be noted that this study was conducted within the context of the Australian healthcare system, where there is a publicly funded universal healthcare insurance scheme. Thus, findings may not be generalisable outside of the Australian context.

## Conclusions

A noteworthy proportion of general practice encounters among women are now for infertility; this and the marked variation in management approaches demonstrated in this study across practices indicate a need for clinical practice guidelines for the management of infertility in the primary care setting. The implementation of clinical guidelines has the potential to improve consistency of care and equity of access across population groups while potentially saving time, money and emotional burden on women and couples.

## supplementary material

10.1136/bmjopen-2024-085149 online supplemental file 1

## Data Availability

Data may be obtained from a third party and are not publicly available.

## References

[1] Cox CM, Thoma ME, Tchangalova N (2022). Infertility prevalence and the methods of estimation from 1990 to 2021: a systematic review and meta-analysis. Hum Reprod Open.

[2] Zegers-Hochschild F, Adamson GD, Dyer S (2017). The International Glossary on Infertility and Fertility Care, 2017. Hum Reprod.

[3] Carson SA, Kallen AN (2021). Diagnosis and Management of Infertility: A Review. JAMA.

[4] Thonneau P, Marchand S, Tallec A (1991). Incidence and main causes of infertility in a resident population (1,850,000) of three French regions (1988-1989). Hum Reprod.

[5] Chooi YC, Ding C, Magkos F (2019). The epidemiology of obesity. Metab Clin Exp.

[6] (2023). Organisation for economic co- operation and development. sf2.3: age of mothers at childbirth and age- specific fertility. https://www.oecd.org/els/soc/SF_2_3_Age_mothers_childbirth.pdf.

[7] Thoma M, Fledderjohann J, Cox C (2021). Oxford Research Encyclopedia of Global Public Health.

[8] Datta J, Palmer MJ, Tanton C (2016). Prevalence of infertility and help seeking among 15 000 women and men. Hum Reprod.

[9] Marino JL, Moore VM, Rumbold AR (2011). Fertility treatments and the young women who use them: an Australian cohort study. Hum Reprod.

[10] Fields E, Chard J, James D (2013). Fertility (update): summary of NICE guidance. BMJ.

[11] Teede HJ, Mousa A, Tay CT (2024). Summary of the 2023 international evidence-based guideline for the assessment and management of polycystic ovary syndrome: an Australian perspective. Med J Aust.

[12] Britt H MG, Bayram C, Henderson J (2016). A Decade of Australian General Practice Activity 2006–07 to 2015–16.

[13] Chambers GM, Harrison C, Raymer J (2019). Infertility management in women and men attending primary care-patient characteristics, management actions and referrals. Hum Reprod.

[14] Charles J, Pan Y, Britt H (2005). Management of infertility in Australian general practice. Aust Fam Physician.

[15] Busingye D, Gianacas C, Pollack A (2019). Data Resource Profile: MedicineInsight, an Australian national primary health care database. Int J Epidemiol.

[16] MedicineInsight (2020). General practice insights report: july 2018– june 2019.

[17] Australian Bureau of Statistics (2021). Socio-Economic Indexes for Areas (SEIFA): Technical Paper. https://www.abs.gov.au/statistics/people/people-and-communities/socio-economic-indexes-areas-seifa-australia/latest-release.

[18] Australian Commission on Safety and Quality in Health Care (2024). MedicineInsight Sydney: ACSQHC. https://www.safetyandquality.gov.au/our-work/indicators-measurement-and-reporting/medicineinsight#practice-reconsent.

[19] Australian Institute of Health and Welfare (2023). Australia’s mothers and babies. https://www.aihw.gov.au/reports/mothers-babies/australias-mothers-babies/contents/demographics-of-mothers-and-babies/key-statistics-and-trends.

[20] Dhalwani NN, Fiaschi L, West J (2013). Occurrence of fertility problems presenting to primary care: population-level estimates of clinical burden and socioeconomic inequalities across the UK. Hum Reprod.

[21] Passet-Wittig J, Greil AL (2021). Factors associated with medical help-seeking for infertility in developed countries: A narrative review of recent literature. Soc Sci Med.

[22] Holowko N, Jones M, Tooth L (2018). Socioeconomic Position and Reproduction: Findings from the Australian Longitudinal Study on Women’s Health. Matern Child Health J.

[23] van Roode T, Sharples K, Dickson N (2017). Life-Course Relationship between Socioeconomic Circumstances and Timing of First Birth in a Birth Cohort. PLoS One.

[24] Zhang C, Harrison C, Britt H (2012). Infertility: management in Australian general practice. Aust Fam Physician.

[25] van der Pluijm-Schouten HW, Hermens RPMG, van Heteren CF (2017). General practitioners’ adherence to work-up and referral recommendations in fertility care. Hum Reprod.

[26] Passet-Wittig J, Greil AL (2021). On estimating the prevalence of use of medically assisted reproduction in developed countries: a critical review of recent literature. *Hum Reprod Open*.

[27] Wyns C, Bergh C (2020). The European IVF-monitoring Consortium for the European Society of Human Reproduction and Embryology. Hum Reprod Open.

[28] Royal College of Obstetricians and Gynaecologists (RCOG) (1999). Guideline Summary No. 2:the initial investigation and management of the infertile couple*. BJU Int.

[29] Morrison C, Bhattacharya S, Hamilton M (2007). Initial management of infertility: an audit of pre-referral investigations and exploration of couples’ views at the interface of primary and secondary care. Hum Fertil (Camb).

[30] Wright M, Hall J, van Gool K (2018). How common is multiple general practice attendance in Australia?. Aust J Gen Pract.

[31] Mina R, Thistlethwaite J, Belcher J (2020). MedicineInsight report: validation of the medicineinsight database: completeness, generalisability and plausibility.

