# Supplementary material for: Trends in clinical encounters and management for infertility among women attending Australian general practice: a national longitudinal study using MedicineInsight, 2011 to 2021
Source: BMJ Open. 2025 Jan 30;15(2):e085149. doi: 10.1136/bmjopen-2024-085149 (PMC11795361; doi:10.1136/bmjopen-2024-085149)
Supplement: online supplemental file 1 [file bmjopen-15-2-s001.docx]

| **Supplementary Table S1. Definitions used to identify infertility consultations and associated clinical management** | | | |
| --- | --- | --- | --- |
| **Infertility Encounters** | **Selected Medications** | **Selected Imaging** | **Selected Pathology** |
| Infertility  Subfertility  Fertility issues  Fertility concerns  Fertility treatment  Anovulation  Fertility – Impaired  Delayed fertility  Fertility referral  Impaired fertility  Fertility problem  Ovulation induction  Difficulty conceiving | Clomifene  Letrozole  Metformin | Ultrasound; pelvis  Hysterosalpingogram  Hystero-salpingo contrast sonography | Anti-Mullerian hormone  Blood Group  DHEAS  FSH  Glucose  Haemoglobin  HBA1C  Hepatitis B serology  Hepatitis C serology  HIV  Hormone assay  Herpes serology  Insulin  LH  Oestradiol  Oestrogen  Progesterone  Prolactin  Rhesus Antibodies  Syphilis  Testosterone  Thyroid function   - Thyroxine - TSH - T3   Varicella serology  Vitamin D |

**Supplemental Figure 1. Practice-level variation in the proportion of infertility clinical encounters where the general practitioner provided specific management, Australia 2011 to 2021**


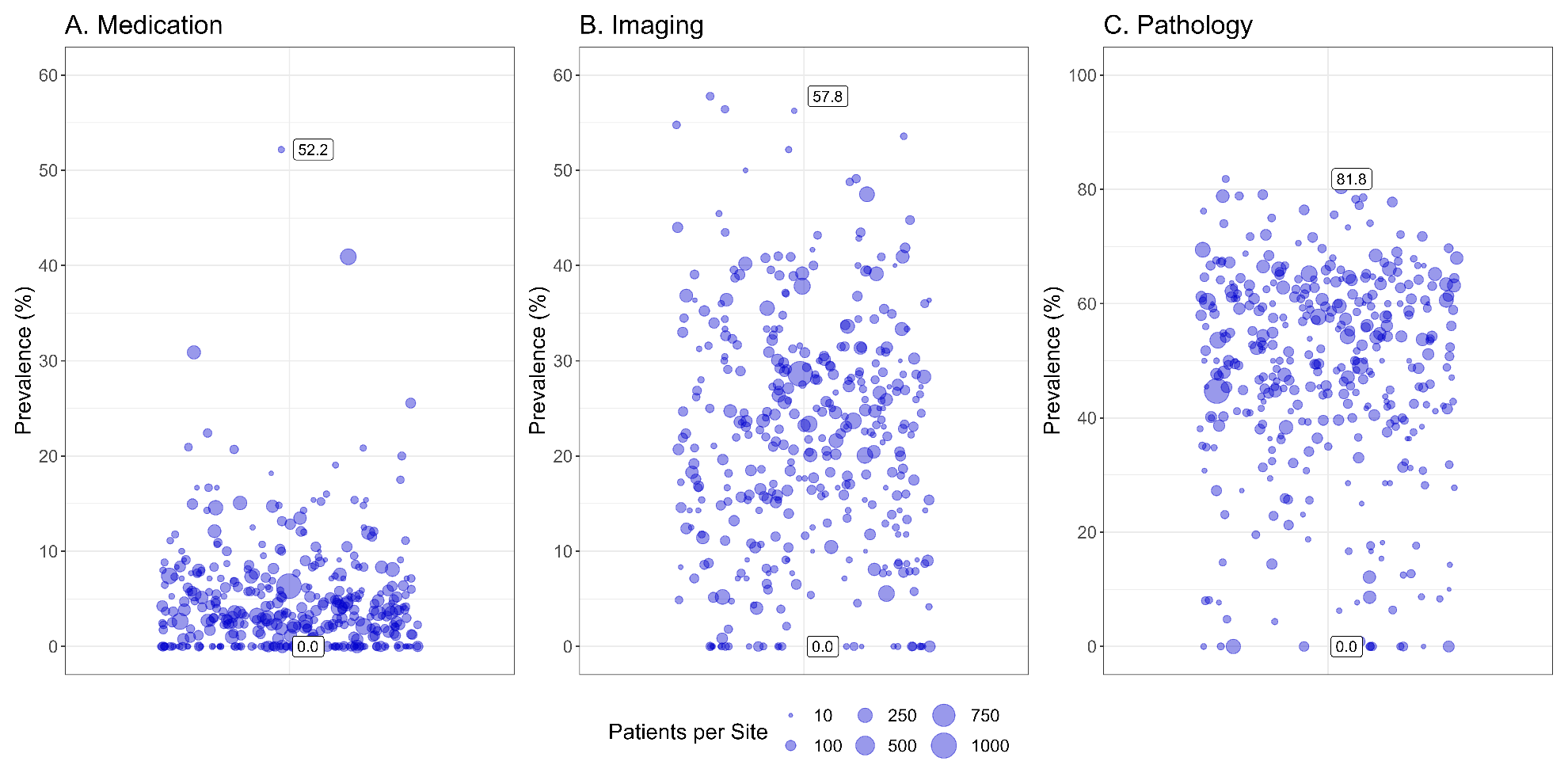


**Supplementary Table S2. Number of women with an infertility encounter prescribed selected medications according to year of first encounter, Australia 2011-2021**

|  |  | | **Year of First Infertility Encounter** | | | | | | | | | | | |
| --- | --- | --- | --- | --- | --- | --- | --- | --- | --- | --- | --- | --- | --- | --- |
|  | **2011** | **2012** | | **2013** | **2014** | **2015** | **2016** | **2017** | **2018** | **2019** | **2020** | **2021** | **P Value*** | **Total** |
| **N** | 1,834 | 1,873 | | 2,159 | 2,254 | 2,533 | 2,584 | 2,695 | 2,824 | 2,969 | 2,928 | 3,018 |  | 27,671 |
| **Any Medication** |  |  | |  |  |  |  |  |  |  |  |  |  |  |
| First Encounter | 64  (3.5) | 71  (3.8) | | 67  (3.1) | 80  (3.6) | 85  (3.4) | 75  (2.9) | 77  (2.9) | 93  (3.3) | 88  (3.0) | 94  (3.2) | 89  (3.0) | 0.011 | 883  (3.2) |
| Any Encounter | 104  (5.7) | 110 (5.9) | | 115 (5.3) | 135 (6.0) | 158 (6.2) | 143 (5.5) | 145 (5.4) | 156 (5.5) | 150 (5.1) | 152 (5.2) | 135 (4.5) | 0.109 | 1,503 (5.4) |
| **Medication Type** |  |  | |  |  |  |  |  |  |  |  |  |  |  |
| Clomifene | 62  (3.4) | 78  (4.2) | | 60  (2.8) | 68  (3.0) | 75  (3.0) | 57  (2.2) | 66  (2.5) | 55  (2.0) | 34  (1.2) | 41  (1.4) | 22  (0.7) | <0.001 | 618  (2.2) |
| Metformin | 56  (3.1) | 41  (2.2) | | 65  (3.0) | 72  (3.2) | 85  (3.4) | 86  (3.3) | 77  (2.9) | 94  (3.3) | 98  (3.3) | 101  (3.5) | 93  (3.1) | 0.162 | 868  (3.1) |
| Letrozole | NR | NR | | 3  (0.1) | 3  (0.1) | 9  (0.4) | 11  (0.4) | 18  (0.7) | 15  (0.5) | 26  (0.9) | 17  (0.6) | 24  (0.8) | <0.001 | 129  (0.5) |

*linear trend test for change in proportion from 2011 to 2021

**Supplemental Table S3. Pathology investigations ordered for female patients attending for infertility related encounter to Australia general practice between January 2011 to December 2021**

|  | **First clinical encounter** | | **Any clinical encounter** | |
| --- | --- | --- | --- | --- |
| **Category** | **Rate per 100**  **(95% CI)** | **OR**  **(95% CI)** | **Rate per 100**  **(95% CI)** | **OR**  **(95% CI)** |
| **Age group** |  |  |  |  |
| 18-24 | 44.9 (42.6, 47.1) | 1.04 (0.94, 1.14) | 52.5 (50.2, 54.7) | 0.96 (0.87, 1.06) |
| 25-29 | 45.8 (44.5, 47.1) | 1.08 (1.01, 1.15) | 55.2 (53.9, 56.5) | 1.07 (1.00, 1.14) |
| 30-34 | 44.0 (42.9, 45.0) | Reference | 53.6 (52.5, 54.6) | Reference |
| 35-39 | 39.2 (38.1, 40.4) | 0.82 (0.77, 0.88) | 48.8 (47.6, 49.9) | 0.82 (0.77, 0.88) |
| 40-44 | 35.1 (33.4, 36.7) | 0.69 (0.63, 0.75) | 42.9 (41.2, 44.6) | 0.65 (0.60, 0.71) |
| 45-49 | 32.7 (29.3, 36.3) | 0.62 (0.53, 0.73) | 38.2 (34.6, 41.9) | 0.54 (0.46, 0.63) |
| **Concession status** |  |  |  |  |
| No concession | 44.2 (43.5, 44.8) | Reference | 53.7 (53.0, 54.4) | Reference |
| Concession holder | 40.1 (38.5, 41.6) | 0.92 (0.86, 0.98) | 48.5 (46.9, 50.1) | 0.89 (0.84, 0.96) |
| Not recorded | 30.0 (28.0, 31.1) | 0.53 (0.49, 0.57) | 36.7 (35.0, 38.3) | 0.50 (0.46, 0.87) |
| **Smoking Status** |  |  |  |  |
| Never Smoker | 45.1 (44.3, 45.9) | Reference | 55.2 (54.4, 56.0) | Reference |
| Ex Smoker | 36.5 (35.2, 37.8) | 0.70 (0.66, 0.75) | 45.0 (44.0, 46.4) | 0.66 (0.62, 0.71) |
| Current Smoker | 37.1 (35.7, 38.4) | 0.72 (0.67, 0.77) | 45.0 (43.6, 46.4) | 0.66 (0.62, 0.71) |
| Not recorded | 43.8 (41.8, 45.7) | 0.94 (0.87, 1.03) | 50.4 (0.48, 0.52) | 0.82 (0.76, 0.90) |
| **Remoteness** |  |  |  |  |
| Major City | 41.4 (40.7, 42.1) | Reference | 51.0 (50.3, 51.7) | Reference |
| Inner/Outer Regional | 43.0 (41.9, 44.1) | 1.07 (1.02, 1.13) | 50.9 (49.7, 52.0) | 0.99 (0.94, 1.05) |
| Remote/Very Remote | 43.6 (37.96, 49.4) | 1.10 (0.87, 1.38) | 49.8 (44.1, 55.6) | 0.95 (0.76, 1.20) |
| Not recorded | 34.1 (25.8, 43.2) | 0.74 (0.51, 1.07) | 41.5 (32.7, 50.7) | 0.68 (0.47, 0.97) |
| **Socioeconomic Status** |  |  |  |  |
| Very Low | 39.9 (38.3, 41.5) | 0.97 (0.89, 1.05) | 46.8 (45.2, 48.4) | 0.86 (0.79, 0.93) |
| Low | 43.3 (41.9, 44.7) | 1.11 (1.03, 1.19) | 51.8 (50.4, 53.3) | 1.05 (0.98, 1.13) |
| Middle | 42.9 (41.6, 44.2) | 1.09 (1.02, 1.17) | 52.1 (50.8, 53.4) | 1.06 (0.99, 1.14) |
| High | 42.3 (41.1, 43.5) | 1.07 (1.00, 1.14) | 52.2 (51.0, 53.4) | 1.07 (1.00, 1.14) |
| Very High | 40.7 (39.6, 41.9) | Reference | 50.6 (49.4, 51.7) | Reference |
| **Indigenous Status** |  |  |  |  |
| Aboriginal and/or TSI | 41.3 (37.4, 45.4) | 0.98 (0.83, 1.15) | 48.5 (44.4, 52.6) | 0.93 (0.76, 1.05) |
| Non-Indigenous | 41.9 (41.3, 42.6) | Reference | 51.4 (50.7, 52.0) | Reference |
| Not recorded | 41.5 (40.1, 42.8) | 0.98 (0.92, 1.04) | 49.4 (48.1, 50.8) | 0.93 (0.87, 1.05) |
| **Asthma** |  |  |  |  |
| No | 42.2 (41.6, 42.8) | Reference | 51.3 (50.7, 51.9) | Reference |
| Yes | 36.8 (34.5, 39.1) | 0.80 (0.72, 0.88) | 45.0 (42.6, 47.4) | 0.78 (0.70, 0.86) |
| **Anxiety** |  |  |  |  |
| No | 42.1 (41.5, 42.7) | Reference | 51.0 (50.4, 51.6) | Reference |
| Yes | 38.3 (36.0, 40.7) | 0.86 (0.77, 0.95) | 49.1 (46.7, 51.6) | 0.93 (0.84, 1.02) |
| **Depression** |  |  |  |  |
| No | 42.3 (41.6, 42.9) | Reference | 51.3 (50.7, 51.9) | Reference |
| Yes | 37.3 (35.3, 39.3) | 0.81 (0.75, 0.89) | 46.6 (44.5, 48.6) | 0.83 (0.76, 0.90) |
| **PCOS** |  |  |  |  |
| No | 42.0 (41.4, 42.6) | Reference | 51.1 (50.5, 51.7) | Reference |
| Yes | 38.1 (35.3, 41.0) | 0.85 (0.75, 0.96) | 46.5 (43.6, 49.4) | 0.83 (0.74, 0.94) |
| **Diabetes** |  |  |  |  |
| No | 41.9 (41.3, 42.5) | Reference | 51.0 (50.4, 51.6) | Reference |
| T1DM | 37.0 (24.3, 51.3) | 0.82 (0.47, 1.42) | 42.6 (29.2, 56.8) | 0.71 (0.42, 1.22) |
| T2DM | 38.2 (29.6, 47.4) | 0.86 (0.60, 1.24) | 44.7 (35.8, 53.9) | 0.78 (0.55, 1.11) |

Abbreviations: OR, odds ratio; CI, confidence interval; TSI, Torres Strait Islander; PCOS, polycystic ovarian syndrome; T1DM, Type 1 Diabetes Mellitus; T2DM, Type 2 Diabetes Mellitus.

**Supplementary Table S4. Number of women with an infertility encounter ordered selected imaging according to year of first encounter, Australia 2011-2021**

|  |  | | **Year of First Infertility Encounter** | | | | | | | | | | | |
| --- | --- | --- | --- | --- | --- | --- | --- | --- | --- | --- | --- | --- | --- | --- |
|  | **2011** | **2012** | | **2013** | **2014** | **2015** | **2016** | **2017** | **2018** | **2019** | **2020** | **2021** | **P Value*** | **Total** |
| **N** | 1,834 | 1,873 | | 2,159 | 2,254 | 2,533 | 2,584 | 2,695 | 2,824 | 2,969 | 2,928 | 3,018 |  | 27,671 |
| **Any Imaging** |  |  | |  |  |  |  |  |  |  |  |  |  |  |
| First Encounter | 185  (10.1) | 240  (12.8) | | 358  (16.6) | 367  (16.3) | 416  (16.4) | 470  (18.2) | 523  (19.4) | 568  (20.1) | 630  (21.2) | 623 (21.3) | 687  (22.8) | <0.001 | 5,067  (18.3) |
| Any Encounter | 239  (13.0) | 308  (16.4) | | 460  (21.3) | 461  (20.5) | 534  (21.1) | 606  (23.5) | 678  (25.2) | 708  (25.1) | 790  (26.6) | 795  (27.2) | 821  (27.2) | <0.001 | 6,400  (23.1) |
| **Imaging Type** |  |  | |  |  |  |  |  |  |  |  |  |  |  |
| Ultrasound; pelvis | 232 (12.7) | 302 (16.1) | | 449 (20.8) | 451 (20.0) | 523 (20.7) | 593 (23.0) | 656 (24.3) | 692 (24.5) | 773 (26.0) | 782 (26.7) | 814 (27.0) | <0.001 | 6,267 (22.7) |
| Hysterosalpingogram | 8  (0.4) | 26 (1.4) | | 23 (1.1) | 13 (0.6) | 14 (0.6) | 15 (0.6) | 41 (1.5) | 28 (1.0) | 30 (1.0) | 16 (0.6) | 13 (0.4) | 0.272 | 227 (0.8) |
| Hystero-salpingo contrast sonography | NR | NR | | 4  (0.2) | 3  (0.1) | 3  (0.1) | 8  (0.3) | 9  (0.3) | 5  (0.2) | 12 (0.4) | 13 (0.4) | 10 (0.3) | <0.001 | 69  (0.3) |

*linear trend test for change in proportion from 2011 to 2021

**Supplementary Table S5. Number of women with an infertility encounter ordered selected pathology tests according to year of first encounter, Australia 2011-2021**

|  |  | | **Year of First Infertility Encounter** | | | | | | | | | | | |
| --- | --- | --- | --- | --- | --- | --- | --- | --- | --- | --- | --- | --- | --- | --- |
|  | **2011** | **2012** | | **2013** | **2014** | **2015** | **2016** | **2017** | **2018** | **2019** | **2020** | **2021** | **P Value*** | **Total** |
| **N** | 1,834 | 1,873 | | 2,159 | 2,254 | 2,533 | 2,584 | 2,695 | 2,824 | 2,969 | 2,928 | 3,018 |  | 27,671 |
| **Any Pathology** |  |  | |  |  |  |  |  |  |  |  |  |  |  |
| First Encounter | 569  (31.0) | 659  (35.2) | | 806  (37.3) | 898  (39.8) | 1,037  (40.9) | 1,052  (40.7) | 1,144  (42.5) | 1,236  (43.8) | 1,391  (46.9) | 1,326  (45.3) | 1,458  (48.3) | <0.001 | 11,576  (41.8) |
| Any Encounter | 681  (37.1) | 793  (42.3) | | 988  (45.8) | 1,057  (46.9) | 1,271  (50.2) | 1,309  (50.7) | 1,403  (52.1) | 1,525  (54.0) | 1,672  (56.3) | 1,651  (56.4) | 1,740  (57.7) | <0.001 | 14,090  (50.9) |
| **Pathology Type** |  |  | |  |  |  |  |  |  |  |  |  |  |  |
| AMH | 41  (2.2) | 101  (5.4) | | 133  (6.2) | 149  (6.6) | 176  (7.0) | 168  (6.5) | 230  (8.5) | 310  (11.0) | 359  (12.1) | 395  (13.5) | 463  (15.3) | <0.001 | 2,525  (9.1) |
| Blood Group | 72  (3.9) | 122  (6.5) | | 133  (6.2) | 173  (7.7) | 182  (7.2) | 197  (7.6) | 217  (8.1) | 289  (10.2) | 295  (9.9) | 305  (10.4) | 340  (11.3) | <0.001 | 2,325  (8.4) |
| DHEAS | 29  (1.6) | 34  (1.8) | | 52  (2.4) | 58  (2.6) | 102  (4.0) | 105  (4.1) | 101  (3.8) | 114  (4.0) | 146  (4.9) | 153  (5.2) | 157  (5.2) | <0.001 | 1,051  (3.8) |
| FSH | 332  (18.1) | 418  (22.3) | | 552  (25.6) | 637  (28.3) | 747  (29.5) | 750  (29.0) | 800  (30.0) | 855  (30.3) | 914  (30.8) | 905  (30.9) | 934  (31.0) | <0.001 | 7,844  (28.4) |
| Glucose | 164  (8.9) | 177  (9.5) | | 242  (11.2) | 287  (12.7) | 361  (14.3) | 409  (15.8) | 413  (15.3) | 453  (16.0) | 553  (18.6) | 501  (17.1) | 565  (18.7) | <0.001 | 4,125  (14.9) |
| Haemoglobin | 410  (22.4) | 513  (27.4) | | 644  (29.8) | 726  (32.2) | 867  (34.2) | 927  (35.9) | 1,008  (37.4) | 1,108  (39.2) | 1,250  (42.1) | 1,243  (42.5) | 1,322  (43.8) | <0.001 | 10,018  (36.2) |
| HBA1C | 12  (0.7) | 11  (0.6) | | 28  (1.3) | 41  (1.8) | 76  (3.0) | 108  (4.2) | 150  (5.6) | 234  (8.3) | 257  (8.7) | 297  (10.1) | 306  (10.1) | <0.001 | 1,520  (5.5) |
| Hep B Serology | 104  (5.7) | 155  (8.3) | | 193  (8.9) | 253  (11.2) | 279  (11.0) | 296  (11.5) | 361  (13.4) | 428  (15.2) | 451  (15.2) | 495  (16.9) | 523  (17.3) | <0.001 | 3,538  (12.8) |
| Hep C Serology | 49  (2.7) | 108  (5.8) | | 133  (6.2) | 173  (7.7) | 194  (7.7) | 208  (8.1) | 278  (10.3) | 332  (11.8) | 342  (11.5) | 386  (13.2) | 401  (13.3) | <0.001 | 2,604  (9.4) |
| HIV | 81  (4.4) | 143  (7.6) | | 177  (8.2) | 231  (10.3) | 260  (10.3) | 272  (10.5) | 338  (12.5) | 405  (14.3) | 411  (13.8) | 473  (16.2) | 504  (16.7) | <0.001 | 3,295  (11.9) |
| Hormone Assay | 3  (0.2) | 3  (0.2) | | NR | 3  (0.1) | 3  (0.1) | NR | 6  (0.2) | 9  (0.3) | 7  (0.2) | 4  (0.1) | 6  (0.2) | 0.180 | 47  (0.2) |
| Herpes Serology | NR | 4  (0.2) | | NR | NR | 3  (0.1) | 5  (0.2) | 7  (0.3) | 6  (0.2) | 10  (0.3) | 10  (0.3) | 3  (0.1) | 0.095 | 54  (0.2) |
| Insulin | 42  (2.3) | 40  (2.1) | | 62  (2.9) | 59  (2.6) | 79  (3.1) | 96  (3.7) | 78  (2.9) | 110  (3.9) | 133  (4.5) | 145  (5.0) | 159  (5.3) | <0.001 | 1,003  (3.6) |
| LH | 324  (17.7) | 413  (22.1) | | 551  (25.5) | 629  (27.9) | 731  (28.9) | 735  (28.4) | 793  (29.4) | 836  (29.6) | 902  (30.4) | 884  (30.2) | 917  (30.4) | <0.001 | 7,715  (27.9) |
| Oestradiol | 184  (10.0) | 223  (11.9) | | 293  (13.6) | 360  (16.0) | 402  (15.9) | 460  (17.8) | 469  (17.4) | 531  (18.8) | 564  (19.0) | 538  (18.4) | 651  (21.6) | <0.001 | 4,675  (16.9) |
| Oestrogen | 55  (3.0) | 112  (6.0) | | 146  (6.8) | 116  (5.2) | 175  (6.9) | 153  (5.9) | 181  (6.7) | 177  (6.3) | 210  (7.1) | 189  (6.5) | 173  (5.7) | 0.002 | 1,687  (6.1) |
| Progesterone | 413  (22.5) | 493  (26.3) | | 623  (28.9) | 687  (30.5) | 782  (30.9) | 830  (32.1) | 889  (33.0) | 934  (33.1) | 984  (33.1) | 929  (31.7) | 1,020  (33.8) | <0.001 | 8,584  (31.0) |
| Prolactin | 197  (10.7) | 277  (14.8) | | 380  (17.6) | 380  (16.9) | 461  (18.2) | 485  (18.8) | 455  (16.9) | 589  (20.9) | 568  (19.1) | 618  (21.1) | 654  (21.7) | <0.001 | 5,064  (18.3) |
| Rhesus Antibodies | 58  (3.2) | 106  (5.7) | | 117  (5.4) | 154  (6.8) | 150  (5.9) | 153  (5.9) | 209  (7.8) | 259  (9.2) | 254  (8.6) | 262  (9.0) | 321  (10.6) | <0.001 | 2,043  (7.4) |
| Syphilis | 47  (2.6) | 97  (5.2) | | 119  (5.5) | 154  (6.8) | 187  (7.4) | 179  (6.9) | 234  (8.7) | 285  (10.1) | 276  (9.3) | 346  (11.8) | 386  (12.8) | <0.001 | 2,310  (8.4) |
| Testosterone | 82  (4.5) | 108  (5.8) | | 171  (7.9) | 207  (9.2) | 260  (10.3) | 318  (12.3) | 327  (12.1) | 408  (14.5) | 453  (15.3) | 459  (15.7) | 500  (16.6) | <0.001 | 3,293  (11.9) |
| Thyroid Function | 474  (25.9) | 559  (29.9) | | 705  (32.7) | 797  (35.4) | 948  (37.4) | 977  (37.8) | 1,062  (39.4) | 1,142  (40.4) | 1,268  (42.7) | 1,310  (44.7) | 1,344  (44.5) | <0.001 | 10,586  (38.3) |
| Varicella Serology | 72  (3.9) | 113  (6.0) | | 141  (6.5) | 215  (9.5) | 258  (10.2) | 260  (10.1) | 300  (11.1) | 368  (13.0) | 390  (13.1) | 440  (15.0) | 455  (15.1) | <0.001 | 3,012  (10.9) |
| Vitamin D | 138  (7.5) | 170  (9.1) | | 222  (10.3) | 275  (12.2) | 300  (11.8) | 297  (11.5) | 356  (13.2) | 452  (16.0) | 514  (17.3) | 567  (19.4) | 577  (19.1) | <0.001 | 3,868  (14.0) |

*linear trend test for change in proportion from 2011 to 2021
