# Supplementary material for: Trends in clinical encounters and management for infertility among women attending Australian general practice: a national longitudinal study using MedicineInsight, 2011 to 2021
Source: BMJ Open. 2025 Jan 30;15(2):e085149. doi: 10.1136/bmjopen-2024-085149 (PMC11795361; doi:10.1136/bmjopen-2024-085149)
Supplement: Supplementary file 2 [file bmjopen-2024-085149.reviewer_comments.pdf]

PEER REVIEW HISTORY

BMJ Open publishes all reviews undertaken for accepted manuscripts. Reviewers are asked to complete a checklist review form and are provided with free text boxes to elaborate on their assessment. These free text comments are reproduced below.

ARTICLE DETAILS

Title (Provisional)

Trends in clinical encounters and management for infertility among women attending Australian general practice: a national longitudinal study using MedicineInsight, 2011 and 2021.

Authors

Fernandez, Renae C; Moore , Vivienne; Boyle, Jacqueline; Rumbold, Alice R.; Davies, Michael; Mazza, Danielle; Grzeskowiak, Luke E

VERSION 1 - REVIEW

**Reviewer**	**1**
**Name**	**Akadri, Adebayo**
**Affiliation**	**Babcock University, Obstetrics and gynaecology**
**Date**	**24-Feb-2024**
**COI**	**None**

I read with great interest the Manuscript titled "Trends in clinical encounters and management for infertility among women attending Australian general practice: a national longitudinal study using MedicineInsight, 2011 and 2021".

The content of the manuscript falls within the scope of the Journal. The content will be of great interest to readers.

The study design is appropriate for the study objective.

The results were presented clearly.

The discussion addressed relevant issues regarding infertility management by GPs in Australia and made valid recommendation regarding the need for clinical practice guidelines for the management of infertility in the primary care setting.

There are however a few concerns that require clarifications

1. The authors used clomiphene and clomifene interchangeably. I suggest that they use one: Clomiphene

2. In discussion, page 20 line 51, the authors wrote “Patterns in the prescription of specific medications by GPs suggest that medications are prescribed to women who may have difficulty accessing specialist care, that is those with lower income, lower socioeconomic status and those living outside major cities.”

The authors should clarify why this is the case since management option is expected to be based on diagnosis. Moreover, the medications prescribed are essentially those used for ovulation induction. Are low income earners more prone to anovulatory infertility?

3. Is there data on diagnosis mage by the GPs?

Overall, the article is interesting and generally well written.

**Reviewer**	**2**
**Name**	**bayuo, jonathan**
**Affiliation**	**Hong Kong Polytechnic University, School of Nursing**
**Date**	**30-Jul-2024**
**COI**	**I have no competing interests.**

The authors present cohort study examining the longitudinal trends in infertility management among women attending general practice. The study is well-written with attention to details and articulating the gap with appropriate methods. Some few comments for your kind consideration:

1. For a study articulating a longitudinal trend, I wonder if logistic regression is the most appropriate statistical test? Not sure though and I have recommended further statistical assessment.

2. The discussion is well raised reflecting the results. Could the authors move a step further to highlight implications of the findings. For instance, with the observed lower rates of infertility among participants who had a commonwealth concession card (low income), current smokers, lived in regional/ remote areas, what are the implications for health service organisation? Adding these implications will greatly sharpen the discussion section. Thank you.

**Reviewer**	**3**
**Name**	**Kelson, Zoe**
**Affiliation**	**University of Exeter, Mathematics**
**Date**	**06-Nov-2024**

COI **None**

This cohort study aims to examine longitudinal trends in infertility management in women attending general practice.

Reviewer comments:

Can the authors please add a description of the methods and statistical analysis in the Abstract?

"The cohort included 2,552,339 women aged 18 to 49 years with one or more general practice clinical encounters between January 2011 to December 2021."

and

"This was a cohort study assembled using data from the National Prescribing Scheme (NPS) MedicineWise MedicineInsight dataset"

and

"MedicineInsight contains electronic health records (EHRs) from approximately 2,700 GP and 662 general practices across Australia (8.2% of all Australian practices). The characteristics of MedicineInsight patients have been previously demonstrated to be nationally representative of the Australian population"

The authors do well to utilise a representative and large dataset for this study.

"Patient characteristics included age (based on year of birth), remoteness, socio-economic indexes for areas (SEIFA), state/territory, Indigenous status, Commonwealth concession card status and smoking status. Women for whom Indigenous status was recorded as unknown or missing were re-categorised as non Indigenous...

Additional characteristics of the cohort included the presence of relevant comorbidities including polycystic ovary syndrome, diabetes, asthma, anxiety and history of depression. Individuals were defined as having any of these clinical conditions based on 'condition flags' included in the MedicineInsight dataset, with the date of recording preceding the date of their first infertility encounter."

and Table 1 footnote: "Missing data: concession status, n=380,146; smoking status, n=643,240, remoteness, n=26,559, indigenous status, n= 804,405 "

Can the authors please comment on whether missing data can be assumed to be missing at random?

Whilst mentioned for Indigenous status, how was missing data handled in the analysis for other characteristics?

Can a sensitivity analysis on the approach to handling missing data please be undertaken?

"We used univariable logistic regression analyses to compare the likelihood of women having a document clinical encounter related to infertility based on individual characteristics. We also used univariable logistic regression analyses to compare the likelihood of women receiving selected clinical management for infertility according to individual characteristics, reported separately according to the time of the first clinical encounter, or any clinical encounter. "

Appropriate modelling methods have been applied.

Can the authors please consider exploring collinearity and correlation between characteristics?

Can multivariate analyses please be performed to assess which characteristics are independently associated with the outcome?

"The annual prevalence for clinical encounters related to infertility increased from 3.35 per 1,000 women in 2011 to 5.67 per 1,000 in 2021. The highest rates were evident among women aged 30-34, with marked increases over time observed among those aged between 30-34, 35-39, and 40-44 (Figure 1). Over the 10-year period, clinical encounters related to infertility increased by 55%, 75% and 111% respectively in these age groups."

and

"At the time of the first encounter, the proportion of women who received at least one specified pathology test increased from 31.0% in 2011 to 48.3% in 2021, and the proportion who received a specified imaging test increased from 10.1% in 2011 to 22.8% in 2021 (Figure 2 a). In contrast, the proportion of women attending encounters resulting in provision of a selected medication was relatively stable from 2011 (3.5%) to 2021 (3.0%). Similar patterns emerged when considering clinical management occurring during any infertility encounter (Figure 2 b). "

and

"Between 2011 and 2021 the proportion of individuals prescribed clomiphene declined from 3.4% to 0.7%, whereas an increase was observed for letrozole (0% to 0.8%) (Supplemental Table S2). Prevalence for metformin remained stable at 3.1%."

and

"The vast majority (>98%) of specified imaging requests involved pelvic ultrasounds, which increased in prevalence from 12.7% in 2011 to 27% in 2021 (Supplemental Table S4)...

Between 2011 and 2021, the largest increases in pathology requests were observed for HbA1C (14-fold: 0.7% to 10.1%) and anti-mullerian hormone (7-fold: 2.2% to 15.3%)."

Can the authors please perform time series and trend analyses to statistically examine the longitudinal nature of the data?

"Lower rates of infertility encounters were evident among those who had a Commonwealth concession card (indicating low income), were current smokers, lived in regional or remote areas or who were Aboriginal and/or Torres Strait Islander (Table 1). In contrast, recorded co-morbidities such as asthma, anxiety, depression, and PCOS were all associated with higher rates of infertility encounters. "

Can statistical outcomes in support of these findings please be reported?

"Table 1. Characteristics of females presenting for infertility encounters to general practice, Australia 2011-2021"

Can the authors please clarify how multiple encounters per woman (with one or more encounters for infertility, and the rest for non-infertility) are treated in this analysis? For instance, if a woman had non-infertility encounters in 2011 and 2015 and an infertility encounter in 2017, how would those cases be reflected in the numerators and denominators seen in this table?

"Table 2. Provision of infertility medications to female patients attending for infertility related encounter to Australia general practice between January 2011 to December 2021"

and

"Table 3. Imaging requests among female patients attending for infertility related encounter to Australia general practice between January 2011 to December 2021"

and

"Supplemental Table S3. Pathology investigations ordered for female patients attending for infertility related encounter to Australia general practice between January 2011 to December 2021"

Can multivariate analyses adjusting for all characteristics simultaneously please additionally be undertaken and reported in these tables?

"Figure 1. Annual prevalence rate of infertility encounters in general practice among female patients aged 18- 49 according to age group, Australia 2011-2021. "

Can uncertainty (e.g. confidence intervals) please be shown on plots?

"Notable limitations of this analysis include the assumption that the selected pathology/imaging were related to the documented encounter reason. We did not examine pathology and imaging history that may have been undertaken for non-infertility reasons. We also don't have data on referrals made to fertility clinics or specialists. However, in countries with existing clinical guidelines, such as the UK and the Netherlands, the recommendation is for basic infertility investigations to be undertaken by GPs prior to referral to specialist care (24, 28), providing the rationale for this study to investigate initial management in primary care. While data are recorded at the individual patient level, patient data is not linked across different general practices. It is therefore possible to double count individuals presenting to different general practices. An Australian survey estimated multiple

general practice attendance at 28%. However, 85% of these individuals also reported having a usual GP, suggesting that attendance at non-usual GPs may be for less complex health concerns (29). MedicineInsight uses a non-random sampling process to recruit the practices, however, the distribution of the sample closely resembles figures from the last Australian census (15). Lastly, it should also be noted that this study was conducted within the context of the Australian health care system, where there is a publicly-funded universal health care insurance scheme. Thus, findings may not be generalisable outside of the Australian context"

The authors acknowledge a number of study limitations.

Can they also please discuss missing data?

"While data are recorded at the individual patient level, patient data is not linked across different general practices. It is therefore possible to double count individuals presenting to different general practices. An Australian survey estimated multiple general practice attendance at 28%"

Is there a way to link these patients across GPs (presumably double medication is non-permissible)?

Is there a method for identifying if a GP is the 'usual GP' or not?

Thanks for providing a copy of the STROBE checklist.

VERSION 1 - AUTHOR RESPONSE

Reviewer: 1
Dr. Adebayo Akadri, Babcock University

Comments to the Author:
I read with great interest the Manuscript titled "Trends in clinical encounters and management for infertility among women attending Australian general practice: a national longitudinal study using MedicineInsight, 2011 and 2021".
The content of the manuscript falls within the scope of the Journal. The content will be of great interest to readers.
The study design is appropriate for the study objective.
The results were presented clearly.
The discussion addressed relevant issues regarding infertility management by GPs in Australia and made valid recommendation regarding the need for clinical practice guidelines for the management of infertility in the primary care setting.
Thank you, Dr Akadri, for your positive and constructive feedback on the manuscript. We provide point by point responses to the concerns raised below.

There are however a few concerns that require clarifications
1. The authors used clomiphene and clomifene interchangeably. I suggest that they use one: Clomiphene.
Thank you for picking up this inconsistency in terms. We have amended the manuscript to consistently use the term 'clomifene' in accordance with the WHO Anatomical Therapeutic Chemical (ATC) Classification .

2. In discussion, page 20 line 51, the authors wrote "Patterns in the prescription of specific medications by GPs suggest that medications are prescribed to women who may have difficulty accessing specialist care, that is those with lower income, lower socioeconomic status and those living outside major cities."
The authors should clarify why this is the case since management option is expected to be based on diagnosis. Moreover, the medications prescribed are essentially those used for ovulation induction. Are low income earners more prone to anovulatory infertility?
We agree with Reviewer 1 that, ideally, clinical management should be based on diagnosis. However, in reality practical constraints mean that many forms of reproductive health care are not available to women who are disadvantaged or do not live in capital cities (this was the subject of a recent Australian Senate Inquiry). Consistent with this understanding, the finding that patients characterised by disadvantage or geographical remoteness are overrepresented among those who obtain clomiphene prescriptions from a general practitioner suggests they face barriers to accessing specialist infertility care and treatments. Low income earners are probably not more prone to anovulatory infertility, they are treated by GPs rather than being referred to services that would be much more expensive or are not available in their locality.

We have added the following to clarify this at Page 20 line 341 - Page 21 line 344.

"Although a universal health care insurance system operates in Australia, the patient out of pocket costs associated with attending a specialised clinic are considerably more than those associated with GP appointments. Medical specialists are also concentrated in major cities. Patterns in the prescription of specific medications by GPs may therefore suggest that medications are prescribed to women who may have difficulty accessing specialist care, that is those with lower income, lower socioeconomic status and those living outside major cities."

3. Is there data on diagnosis mage by the GPs?
The MedicineInsight dataset contains validated diagnoses flags for medical conditions including asthma, diabetes, depression, anxiety, and PCOS which have all been included as participant characteristics. Unfortunately, there are no validated diagnoses flags specific to infertility.

Overall, the article is interesting and generally well written.
Thank you.

Reviewer: 2
Mr. jonathan bayuo, Hong Kong Polytechnic University

Comments to the Author:
The authors present cohort study examining the longitudinal trends in infertility management

among women attending general practice. The study is well-written with attention to details and articulating the gap with appropriate methods.
Thank you, Dr Bayuo, for your positive and constructive feedback on the manuscript. We provide point by point responses to the concerns raised below.

Some few comments for your kind consideration:

1. For a study articulating a longitudinal trend, I wonder if logistic regression is the most appropriate statistical test? Not sure though and I have recommended further statistical assessment.
Thank you for this comment. Statistical assessment by Reviewer 3 below (Reviewer 3, Comment 4) indicates that appropriate modelling methods have been applied.

2. The discussion is well raised reflecting the results. Could the authors move a step further to highlight implications of the findings. For instance, with the observed lower rates of infertility among participants who had a commonwealth concession card (low income), current smokers, lived in regional/ remote areas, what are the implications for health service organisation? Adding these implications will greatly sharpen the discussion section. Thank you.
The reviewer seems to be referring to the lower rates of infertility <u>encounters</u> among participants with concession card etc (we did not have information on rates of infertility). The overriding implication for health service organisation for these patients is the provision of primary healthcare services to meet overall needs, of which fertility treatment is just one component. This is an example of the "Inverse Care Law" (Harris & Harris 2022).

We have added the following to the discussion at Page 20 line 318-320 of the manuscript:

"The overriding implication for health service organisation for these patients is that there is a need for provision of primary healthcare services to meet overall needs, of which fertility treatment is just one component."

Reference: Harris E, Harris MF. (2023) An exploration of the inverse care law and market forces in Australian primary health care. Australian Journal of Primary Health 29, 137-141.

Reviewer: 3
Prof. Zoe Kelson, University of Exeter
Comments to the Author:
This cohort study aims to examine longitudinal trends in infertility management in women attending general practice.
Thank you, Prof Kelson, for your thorough review and constructive feedback on our paper. Please point by point responses below.

Reviewer comments:

1. Can the authors please add a description of the methods and statistical analysis in the Abstract?
Thank you for this suggestion. We have updated the abstract to include details of the methods and statistical analysis under the 'Primary and Secondary Outcome Measures' heading. Please refer to the revised abstract on Page 3 Line 40-47.

2. "The cohort included 2,552,339 women aged 18 to 49 years with one or more general practice clinical encounters between January 2011 to December 2021."
and
"This was a cohort study assembled using data from the National Prescribing Scheme (NPS) MedicineWise MedicineInsight dataset"
and
"MedicineInsight contains electronic health records (EHRs) from approximately 2,700 GP and 662 general practices across Australia (8.2% of all Australian practices). The characteristics of MedicineInsight patients have been previously demonstrated to be nationally representative of the Australian population"
The authors do well to utilise a representative and large dataset for this study.
Thank you.

3. "Patient characteristics included age (based on year of birth), remoteness, socio-economic indexes for areas (SEIFA), state/territory, Indigenous status, Commonwealth concession card status and smoking status. Women for whom Indigenous status was recorded as unknown or missing were re-categorised as non Indigenous...
Additional characteristics of the cohort included the presence of relevant comorbidities including polycystic ovary syndrome, diabetes, asthma, anxiety and history of depression. Individuals were defined as having any of these clinical conditions based on 'condition flags' included in the MedicineInsight dataset, with the date of recording preceding the date of their first infertility encounter."
and Table 1 footnote: "Missing data: concession status, n=380,146; smoking status, n=643,240, remoteness, n=26,559, indigenous status, n= 804,405 "
Can the authors please comment on whether missing data can be assumed to be missing at random?

Missing data is not missing at random. Data are more likely to be complete for those who are regular patients of a general practice, compared with someone who might have visited a general practice on just a single occasion (Busingye et al. 2020).

Because covariates like concessional status, smoking status, and Indigenous status rely on the General Practitioner selecting "yes" for the presence of the variable, it is highly likely that where nothing is recorded it is suggestive of the individual not having the characteristic of interest.

We have addressed this in the manuscript by providing results for the "not recorded" category for each of these covariates (concessional status, smoking status, remoteness, indigenous status) in tables 1, 2, 3 and supplementary table S3. We have also explained the implications of these results in the limitations section of the discussion (Page 23 Line 395-405) as requested below at comment #10.

Reference: Busingye D, Myton R, Mina R, Thistlethwaite J, Belcher J, Chidwick K. MedicineInsight report: Validation of the MedicineInsight database: completeness, generalisability and plausibility. Sydney: NPS MedicineWise, 2020

Whilst mentioned for Indigenous status, how was missing data handled in the analysis for other characteristics?
This was an unfortunate error in the manuscript. Data that are not recorded are now reported separately under each category and we have provided outcome estimates for each of these in the tables.

Can a sensitivity analysis on the approach to handling missing data please be undertaken?
As explained above, we have now included "not recorded" as a separate row under each covariate.

4. "We used univariable logistic regression analyses to compare the likelihood of women having a document clinical encounter related to infertility based on individual characteristics. We also used univariable logistic regression analyses to compare the likelihood of women receiving selected clinical management for infertility according to individual characteristics, reported separately according to the time of the first clinical encounter, or any clinical encounter. "
Appropriate modelling methods have been applied.
Can the authors please consider exploring collinearity and correlation between characteristics?
Variables such as having a concession card and smoking are known to be associated, but not to the degree that creates problems with collinearity, and all of our regression models converged (unaffected by collinearity).

Can multivariate analyses please be performed to assess which characteristics are independently associated with the outcome?
The purpose of this paper is not to identify patient characteristics independently associated with the outcome of clinical encounter related to infertility. We aim to describe relevant patient characteristics not to determine a hierarchy (that splits effect estimates in a way that may be misleading, see Green & Popham 2019). Therefore, we have not used mutually adjusted models.

Reference: Green, M.J., Popham, F. Interpreting mutual adjustment for multiple indicators of socioeconomic position without committing mutual adjustment fallacies. BMC Public Health 19, 10 (2019). https://doi.org/10.1186/s12889-018-6364-y

5. "The annual prevalence for clinical encounters related to infertility increased from 3.35 per 1,000 women in 2011 to 5.67 per 1,000 in 2021. The highest rates were evident among women aged 30-34, with marked increases over time observed among those aged between 30-34, 35-39, and 40-44 (Figure 1). Over the 10-year period, clinical encounters related to infertility increased by 55%, 75% and 111% respectively in these age groups."
and
"At the time of the first encounter, the proportion of women who received at least one specified pathology test increased from 31.0% in 2011 to 48.3% in 2021, and the proportion who received a specified imaging test increased from 10.1% in 2011 to 22.8% in 2021 (Figure 2 a). In contrast, the proportion of women attending encounters resulting in provision of a selected medication was relatively stable from 2011 (3.5%) to 2021 (3.0%). Similar patterns emerged when considering clinical management occurring during any infertility encounter (Figure 2 b). "
and
"Between 2011 and 2021 the proportion of individuals prescribed clomiphene declined from 3.4% to 0.7%, whereas an increase was observed for letrozole (0% to 0.8%) (Supplemental Table S2).

Prevalence for metformin remained stable at 3.1%."
and
"The vast majority (>98%) of specified imaging requests involved pelvic ultrasounds, which increased in prevalence from 12.7% in 2011 to 27% in 2021 (Supplemental Table S4)...
Between 2011 and 2021, the largest increases in pathology requests were observed for HbA1C (14-fold: 0.7% to 10.1%) and anti-mullerian hormone (7-fold: 2.2% to 15.3%)."

Can the authors please perform time series and trend analyses to statistically examine the longitudinal nature of the data?
P-Values for analysis of trends have now been included in the relevant tables (Supplementary tables S2, S4, S5). We have added relevant p-values to the manuscript text in the results section.

We have also added information to the methods (Page 10 Line 222-224):
"Longitudinal trends in the proportion of women receiving selected clinical management for infertility were analysed by including year as a continuous variable in the logistic regression model."

6. "Lower rates of infertility encounters were evident among those who had a Commonwealth concession card (indicating low income), were current smokers, lived in regional or remote areas or who were Aboriginal and/or Torres Strait Islander (Table 1). In contrast, recorded co-morbidities such as asthma, anxiety, depression, and PCOS were all associated with higher rates of infertility encounters. "
Can statistical outcomes in support of these findings please be reported?
The statistical outcomes (odds ratios and 95% confidence intervals) in support of these findings are all in Table 1 and we have tried to make that clearer in the text Page 11, Line 241 – "(Table 1)" added after "encounters". (If the editors wish we can add odds ratios to this sentence, but that is not consistent the way we have summarised results throughout, preferring not to repeat detail that is in the tables/figures.)

7. "Table 1. Characteristics of females presenting for infertility encounters to general practice, Australia 2011-2021"
Can the authors please clarify how multiple encounters per woman (with one or more encounters for infertility, and the rest for non-infertility) are treated in this analysis? For instance, if a woman had non-infertility encounters in 2011 and 2015 and an infertility encounter in 2017, how would those cases be reflected in the numerators and denominators seen in this table?
For those with an infertility encounter, the characteristics reflect those at the time of the infertility encounter. For those without an infertility encounter, the characteristics reflect those at the time of their most recent encounter within the study period.

In light of data on rates by age group being presented in Figure 1, we have removed data on age from Table 1 to avoid unnecessary duplication.

8. "Table 2. Provision of infertility medications to female patients attending for infertility related encounter to Australia general practice between January 2011 to December 2021"
and
"Table 3. Imaging requests among female patients attending for infertility related encounter to

Australia general practice between January 2011 to December 2021"
and
"Supplemental Table S3. Pathology investigations ordered for female patients attending for infertility related encounter to Australia general practice between January 2011 to December 2021"
Can multivariate analyses adjusting for all characteristics simultaneously please additionally be undertaken and reported in these tables?
Please see above response to the same point made in comment #4.

9. "Figure 1. Annual prevalence rate of infertility encounters in general practice among female patients aged 18- 49 according to age group, Australia 2011-2021. "
Can uncertainty (e.g. confidence intervals) please be shown on plots?
Confidence intervals have now been added to each of the plots (Fig 1, Fig 2a & b).

10. The authors acknowledge a number of study limitations. Can they also please discuss missing data?

We have now added results for the not recorded (missing) category in the tables. We have added the following paragraph and a relevant reference to the limitations section to explain that missingness is strongly correlated with being a new patient to the general practice, which explains lower prevalence of presenting for infertility and receiving clinical management. Please see Page 23 Lines 395-405.

"A considerable proportion of data missing data for the concession card, smoking and indigenous status covariates was not recorded. These data are not likely to be missing at random. Data entry often relies on GPs selecting "yes" to indicate the presence of the characteristic, thus it is highly likely that where nothing is recorded it is suggestive of the individual not having the characteristic of interest. Further, the strongest predictor of missing data in the MedicineInsight dataset is the number of clinical encounters with the general practice site, with greater missing data for those with fewer clinical encounters (31). As statistical imputation would not be appropriate in this case, we present regression results for a "not recorded" category. Women for who data on these covariates was not recorded were less likely to attend for an infertility consultation or receive clinical management for infertility. This is not unexpected, as given the sensitivity and complexity of infertility, women may be less inclined to see a new or temporary GP for this purpose."

Busingye D, Myton R, Mina R, Thistlethwaite J, Belcher J, Chidwick K. MedicineInsight report: Validation of the MedicineInsight database: completeness, generalisability and plausibility. Sydney: NPS MedicineWise, 2020

11. "While data are recorded at the individual patient level, patient data is not lingked across different general practices. It is therefore possible to double count individuals presenting to different general practices. An Australian survey estimated multiple general practice attendance at 28%" [Quote from manuscript]
Is there a way to link these patients across GPs (presumably double medication is non-permissible)?
Is there a method for identifying if a GP is the 'usual GP' or not?
We are not able to link patients across GPs nor can we identify whether a GP is the usual GP or not. Infertility medication does not have the incentives that would mean patients might be inclined to "doctor shop" (as is known to occur for pain medications).

VERSION 2 - REVIEW

**Reviewer**	**1**
**Name**	**Akadri, Adebayo**
**Affiliation**	**Babcock University, Obstetrics and gynaecology**
**Date**	**15-Dec-2024**
**COI**	

The authors have addressed the concerns that were earlier highlighted.
